# Methotrexate for Cornual Ectopic Pregnancy

**DOI:** 10.7759/cureus.9642

**Published:** 2020-08-10

**Authors:** Barbara M Parker, Anupam K Gupta, Anastasios Lymperopoulos, John Parker

**Affiliations:** 1 Clinical Pharmacy, AdventHealth Orlando and Rockledge Regional Medical Center, Orlando, USA; 2 Minimally Invasive Surgery, University of Miami Hospital, Miami, USA; 3 Pharmacology, Nova Southeastern University, Fort Lauderdale, USA; 4 Obstetrics and Gynecology, Adventhealth Altamonte, Altamonte Springs, USA

**Keywords:** methotrexate, cornual pregnancy

## Abstract

A 27-year-old pregnant female presented to the emergency department with pelvic pain and vaginal bleeding. At the time of diagnosis, she had an ectopic pregnancy in the right cornua 4 cm in size with a human chorionic gonadotropin (hCG) value of 14,438 international units per mL. The ectopic pregnancy was initially managed by intramuscular methotrexate. However, after 10 days, despite an hCG value drop to 409 international units per mL, an indication that methotrexate was working, the patient had a subsequent rupture with hemoperitoneum necessitating exploratory laparotomy and abdominal hysterectomy.

## Introduction

Ectopic pregnancy is a pregnancy outside of the uterine cavity. In about 4% of ectopic pregnancy, it can involve the cornua [1-6]. Cornual pregnancy is a rare type of ectopic pregnancy in which the embryo implants in the junction between the fallopian tube and the uterus [4,7]. Diagnosis is challenging because on ultrasound, the pregnancy often appears to be intrauterine. Vessels leading from the cornua laterally may help with proper diagnosis [7]. Methotrexate (MTX) is used widely as the primary form of treatment for ectopic pregnancy [1,8,9]. In many instances, it has been successfully employed in the treatment of tubal ectopic pregnancies, but for an interstitial location it is used less commonly and unsuccessful cases have also been reported [3,10-12]. The purpose of this case report is to report a case where MTX was administered intramuscularly as initial treatment, but due to complications and rupture of the right posterior cornu and risk of hemorrhage, total abdominal hysterectomy was required.

## Case presentation

**Figure 1:**
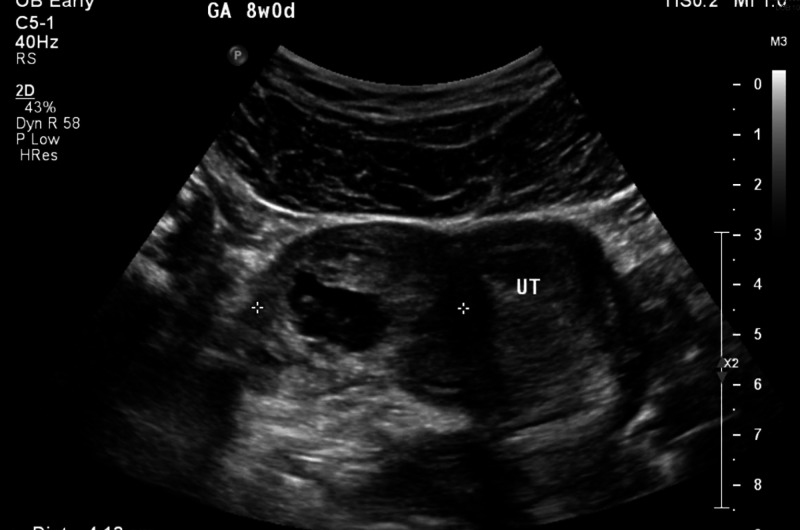
Day 1 ultrasonography (US) of cornual ectopic pregnancy in relation to the uterus (UT)

A 27-year-old African American female gravida 1 (G1) para 0 (P0) at seven weeks, four days, weight 81.3 kg, presented to the emergency department with pelvic pain and vaginal bleeding for two days. Her serum beta-human chorionic gonadotropin (hCG) came to be 14,438 IU/mL (normal reference values: <5 IU/mL non-pregnant, >25 IU/mL pregnant). Ultrasonography revealed a 3.7 x 4.3 x 3.2 cm thick echogenic round mass, a central sonolucent appearance with an apparent 12-mm fetal pole with no cardiac activity. The non-viable embryo was located in close proximity to the right fundal uterine wall consistent with a cornual ectopic pregnancy (Figure 1). Her past medical history included congenital HIV and post-traumatic stress disorder. She had no past surgical history. Her current medications included a prenatal vitamin and highly active antiretroviral therapy (HAART), in the form of Biktarvy by mouth once daily. Significant labs upon admission included a hemoglobin of 12.1 g/dL (normal reference value: 12-15.5g/dL) with a hematocrit of 36.7% (normal reference value: 37%-48%). She was hemodynamically stable. MTX was recommended to halt trophoblastic cell growth. This strategy is generally effective and is a fertility sparing method for treating unruptured cornual pregnancies [4]. A risk versus benefit analysis was discussed with the patient. Alternative treatments including surgery were compared and contrasted. The patient expressed understanding and desired MTX treatment. She was also advised to discontinue her prenatal vitamin. An MTX dose of 50 mg/m^2^ or 100 mg total was administered intramuscularly in divided doses. The patient was stable after discharge from the hospital and was prescribed hydrocodone/acetaminophen 5/325 mg every six hours as needed for abdominal pain for seven days, and scheduled for follow-up.

**Figure 2:**
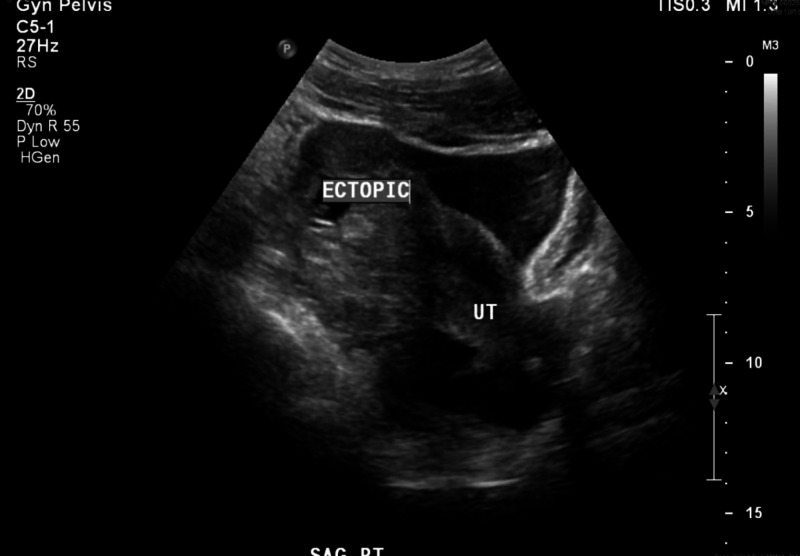
Day 10 ultrasonography (US) of cornual ectopic pregnancy rupture with hemoperitoneum

Ten days after receiving MTX treatment, the patient presented again to the emergency department with the development of severe abdominal pain. Her hemoglobin and hematocrit had fallen to 9.9 g/dL and 30% lower than her baseline. Her quantitative beta-hCG level had decreased to 409 IU/mL. The patient had a surgical abdomen with peritoneal signs. She was unable to position the right side of her pelvis on the bed due to extreme pain. Uterine rupture was suspected secondary to abnormal pregnancy. Repeat ultrasonography was consistent with a right cornual ectopic pregnancy with likely hemoperitoneum (Figure 2). The patient was urgently taken to the operating room for diagnostic laparoscopy that was converted to laparotomy. General endotracheal anesthesia was administered. After elevation of the uterus and inspection of the whole, there were no clear margins for wedge resection and closure of a visible necrotic aperture on the right portion of the uterus was not possible. The patient's estimated blood loss was 500 mL, all of which was evacuated hemoperitoneum with irrigation. The situation was discussed with the patient's family, and a decision was made to proceed with hysterectomy due to the risks of massive hemorrhage. The patient underwent a total abdominal hysterectomy, bilateral salpingectomy, and diagnostic cystoscopy.

## Discussion

Cornual ectopic pregnancy (interstitial pregnancy) is a rare type of ectopic pregnancy that occurs in roughly 2%-4% of ectopic pregnancies and 1/2,500-1/5,000 of all pregnancies [1-6]. The embryo embeds itself into the funnel-shaped area where the uterus connects to the fallopian tubes [1]. Cornual pregnancies pose a diagnostic and therapeutic challenge with significant high morbidity and mortality (2.2%-2.5%) and are associated with massive intraperitoneal bleeding during the first trimester [2,3,5,13]. Without early and rapid diagnosis and treatment, the gestational sac grows to cause uterine rupture and bleeding [2,14]. Cornual ectopic pregnancies, like other ectopic pregnancies, include a high level of serum beta-hCG, persistent light vaginal bleeding despite amenorrhea, and sometimes early pelvic pain [2]. Increased incidence of cornual ectopic pregnancy is associated with assisted reproductive technologies, previous salpingectomy or other tubal surgery, rudimentary horn, history of infections of the reproductive tract, or previous tubal pregnancy and proximal intratubal adhesions. Location makes early diagnosis difficult.

Cornual ectopic pregnancy ultrasonographic criteria include a gestational sac separate from the uterine cavity or empty uterine cavity with thin endometrium (less than 5 mm) around the gestational sac [2-4]. An echogenic line may also be seen in the central endometrial cavity that extends until the gestational sac [2,4].

Traditionally, cornual resection and hysterectomy were the preferred surgical treatments; however, future fertility is affected [1,13,14]. Successful management includes early ultrasonographic diagnosis, laparoscopic resection, and suturing of the uterine cornua [2]. Conservative management treatment goals of an ectopic pregnancy include to halt the development and growth of the embryo, allow resorption of the gestational sac, and maintain the patient’s future fertility [8]. MTX is a chemotherapeutic agent that arrests the growth of the trophoblast by inhibiting DNA synthesis [15,16]. MTX is the most common conservative treatment for the management of early ectopic pregnancy with a success rate of 91% and up to 66.7% in cornual ectopic [1,8,9]. Women desiring to preserve their fertility may consider systemic MTX or intralesional and or local MTX [1,10]. Systemic MTX has greater maternal risk, including higher morbidity and mortality, and systemic side effects (e.g hematologic or hepatic) versus local therapy [1].

The success rate of primary treatment with MTX by several studies reaches between 74% and 100% [4,15-17]. Medical treatment with MTX is less effective when the gestational age is >9 weeks, when the beta-hCG level is >10,000 mIU/mL, when fetal cardiac activity is present, and when the crown-rump length is >10 mm [8,15]. This rate depends greatly on serum beta-hCG levels: the less it is, the greater the chance for ectopic pregnancy to disappear completely [16]. For example, in one set of studies, a success rate of 88% was associated with intramuscular MTX when beta-hCG was less than 5,000 IU/mL and gestational sac diameter was less than 3 cm [16,18]. A slightly higher success rate (with increased therapeutic levels of the drug at the site of administration) and reduced side effects from local administration have been reported [3,16]. In addition, the method offers faster control of trophoblast growth and is more cost effective than surgery [10,16]. A retrospective study of direct MTX injection into the gestational sac under ultrasound visualization in 14 hemodynamically stable patients with interstitial pregnancy showed this technique as an effective alternative to surgery [3].

Choice of treatment depends mostly on clinical situation, stability of the patient's condition, and expertise [2,4]. Ideally in this case, after the failure of MTX, had the patient's condition remained stable, the next step would be to wedge resect the ectopic pregnancy and cornual region of the uterus in order to preserve uterine integrity [8]. Our patient had a beta-hCG level >10,000 mIU/mL with a 12 mm crown-rump length, which may explain the failure of MTX. However, in one reported case of cornual ectopic, local MTX was used successfully and this patient had a crown rump greater than 10 mm as well but met the above b-HCG specifications [3]. A 36-year-old patient had a beta-hCG of 5,055 mIU/mL with a gestational sac of 12 x 11 mm in diameter, and after 24 days post-MTX treatment, her beta-hCG was undetectable and she was able to conceive 12 weeks later. In patients with the ruptured uterus or with hypovolemic shock, laparostomy, cornual resection, and hysterectomy are employed [4]. In a reported 53 cases of cornual ectopic pregnancy that were managed with laparoscopic surgery, the estimated blood loss was approximately 500 mL in most cases, consistent with our patient case [13].

## Conclusions

Determining an upper limit of beta-hCG value at which medical treatment with MTX will fail is yet to be clear. Various injected doses of MTX of 12.5, 25, and 100 mg have been provided in the literature with higher doses needed for higher hCG values to avoid therapy failure. Surgical treatment requires advanced laparoscopic skills and technique to manage or avoid uterine hemorrhage and to reconstruct the cornua. Conservative management with MTX remains preferred for cornual ectopic pregnancies, but it is crucial to bear in mind surgical intervention may still be required down the line given patient response and individual characteristics.
